# A Smartphone App (mDASHNa-CC) to Support Healthy Diet and Hypertension Control for Chinese Canadian Seniors: Protocol for Design, Usability and Feasibility Testing

**DOI:** 10.2196/15545

**Published:** 2020-04-02

**Authors:** Ping Zou, Jennifer Stinson, Monica Parry, Cindy-Lee Dennis, Yeqin Yang, Zhongqiu Lu

**Affiliations:** 1 School of Nursing Nipissing University Toronto, ON Canada; 2 Lawrence Bloomberg Faculty of Nursing, University of Toronto Hospital for Sick Children Toronto, ON Canada; 3 Lawrence Bloomberg Faculty of Nursing, University of Toronto Toronto, ON Canada; 4 Lawrence Bloomberg Faculty of Nursing and Department of Psychiatry, University of Toronto Toronto, ON Canada; 5 School of Nursing, Wenzhou Medical University Wenzhou China

**Keywords:** diet, hypertension, smartphone app, senior, Chinese, Canada

## Abstract

**Background:**

This proposed study aims to translate the Dietary Approach to Stop Hypertension with Sodium (Na) Reduction for Chinese Canadians (DASHNa-CC), a classroom-based, antihypertensive, dietary educational intervention, to an innovative smartphone app (mDASHNa-CC). This study will enable Chinese Canadian seniors to access antihypertensive dietary interventions anytime, regardless of where they are. It is hypothesized that senior Chinese Canadians will be satisfied with their experiences using the mDASHNa-CC app and that the use of this app could lead to a decrease in their blood pressure and improvement in their health-related quality of life.

**Objective:**

The goal of this study is to design and test the usability and feasibility of a smartphone-based dietary educational app to support a healthy diet and hypertension control for Chinese Canadian seniors.

**Methods:**

A mixed-method two-phase design will be used. The study will be conducted in a Chinese immigrant community in Toronto, Ontario, Canada. Chinese Canadian seniors, who are at least 65 years old, self-identified as Chinese, living in Canada, and with elevated blood pressure, will be recruited. In Phase I, we will design and test the usability of the app using a user-centered approach. In Phase II, we will test the feasibility of the app, including implementation (primary outcomes of accrual and attrition rates, technical issues, acceptability of the app, and adherence to the intervention) and preliminary effectiveness (secondary outcomes of systolic and diastolic blood pressure, weight, waist circumference, health-related quality of life, and health service utilization), using a pilot, two-group, randomized controlled trial with a sample size of 60 participants in a Chinese Canadian community.

**Results:**

The study is supported by the Startup Research Grant from Nipissing University, Canada. The research ethics application is under review by a university research ethics review board.

**Conclusions:**

The study results will make several contributions to the existing literature, including illustrating the rigorous design and testing of smartphone app technology for hypertension self-management in the community, exploring an approach to incorporating traditional medicine into chronic illness management in minority communities and promoting equal access to current technology among minority immigrant senior groups.

**Trial Registration:**

Clinicaltrials.gov NCT03988894; https://clinicaltrials.gov/ct2/show/NCT03988894

**International Registered Report Identifier (IRRID):**

PRR1-10.2196/15545

## Introduction

### Rationale for the Intervention

In Canada, 1.3 million Chinese individuals comprise approximately 4.0% of Canada’s population and 21.2% of the country’s visible minorities [1]. In this Chinese population, hypertension is the most prominent risk factor for cardiovascular disease and accounts for a large proportion of stroke [2] and heart failure [3]. With a 15.1% hypertension prevalence rate [4], Chinese Canadians are at an increased risk of cardiovascular disease and associated morbidity and mortality. Because the prevalence of hypertension increases with age, Chinese Canadian seniors are at especially high risk for hypertension and related mortality [4].

Compared with antihypertensive dietary suggestions in hypertension care guidelines [5], Chinese Canadians have a suboptimal dietary intake, which impacts their blood pressure control. Chinese Canadians’ sodium intake is higher than antihypertensive dietary recommendations [6], and a high proportion of Chinese Canadians consume fewer fruits and vegetables than antihypertensive dietary recommendations [7,8]. In addition, Chinese Canadians have a low dairy intake compared with antihypertensive dietary recommendations [9]. Dietary factors have been identified as the most important risk factors for hypertension among the Chinese population [10]. As such, effective dietary interventions are needed to assist with blood pressure control in Chinese Canadian seniors [11,12].

### Dietary Interventions for Hypertension Control

The Dietary Approach to Stop Hypertension (DASH) and sodium reduction are antihypertensive dietary interventions recommended by Canadian hypertension care guidelines [5,13]. Focused on healthy dietary patterns rather than specific nutrients, the DASH diet includes eight food groups (grains, vegetables, fruits, meats, dairy, nuts, fats, and sweets), with specific serving suggestions [14]. The DASH studies demonstrate an effective systolic blood pressure reduction of 4-11 mmHg and a diastolic blood pressure reduction of 2-6 mmHg [15-19]. A systematic review has suggested that sodium reduction is also related to decreased blood pressure [20]. However, the DASH and sodium reduction interventions do not consider the psychosocial factors that influence dietary behaviors, nor have they been tested among Chinese Canadians in a community setting [5,15]. A review paper explored the cultural factors influencing diet and hypertension control in the Chinese Canadian population and suggested that English language proficiency, health literacy, traditional Chinese diet, migration and acculturation, and traditional Chinese medicine (TCM) influence Chinese Canadians' dietary practices [21]. A culturally tailored intervention is thus needed to facilitate blood pressure control in Chinese Canadians.

### Incorporation of Traditional Chinese Medicine for Hypertension Control

Chinese Canadians rely strongly on TCM for chronic illness management and prefer to incorporate TCM into their health care [11]. Food therapy is an essential component of TCM and has been acknowledged as a successful therapy for more than 3000 years [22]. In TCM, food is conceptualized with both nutritional and functional considerations. Like medicines, food can be used to maintain health, prevent and treat diseases, and facilitate rehabilitation [23-27]. There are four principles of TCM food therapy, including light eating, balancing the hot and cold nature of food, harmony of the five flavors of food, and consistency of diets with different health conditions [28,29]. The principal investigator of this study has previously published a literature review on TCM food therapy and hypertension control using a rigorous review method and statistical analysis [30]. Findings suggest that some foods have antihypertensive functions [31-41], and food therapy can facilitate hypertension control [42-45].

### Antihypertensive Diet Apps for the Chinese Population

We conducted a scoping review of existing antihypertensive diet apps written in Chinese on the current market. We searched in various app stores and found 24 apps (15 on iTunes, seven on the Google Play Store, and two on the Chinese App Market). All apps were written in Chinese and focused on diets for hypertension control. We screened the app description and conducted a content analysis. We identified several gaps, including: (1) the app producers had no credentials from any licensed health care professional or research team; (2) all apps were in electronic book form, only providing information with no user interactivity; (3) none of the apps focused on senior users; and (4) most apps from iTunes and the Google Play Store were based on Western diets, which may not fit with Chinese diets. The findings from our review are consistent with a review of app studies on hypertension control, which stated that most of the current apps lack standardization and scientific validation [46]. The mobile Dietary Approach to Stop Hypertension with Sodium (Na) Reduction for Chinese Canadians (mDASHNa-CC) app differs from other related apps in the current market in that it will be developed by a team of health care professionals, involve community end-users in the development process, be based on the current gold standard of antihypertensive dietary interventions, incorporate TCM to ensure that it is culturally significant for Chinese seniors, provide immediate response and recommendations according to the patients’ current conditions, and be scientifically tested by a Randomized Control Trial.

### Preliminary Work: Success of the DASHNa-CC Pilot Trial

The Dietary Approach to Stop Hypertension with Sodium Reduction for Chinese Canadians (DASHNa-CC) intervention was designed based on current literature and clinical expertise. The DASHNa-CC integrates TCM food therapy into the DASH and sodium reduction diary intervention for blood pressure control. Adapted from DASH, DASHNa-CC is designed as a standardized, culturally sensitive, dietary education intervention for Chinese Canadians. The contents of the DASHNa-CC intervention have three components: (1) the DASH diet pattern, including characteristics of the DASH diet, serving size estimation tool, foods rich in calcium, and foods rich in potassium; (2) sodium reduction, including the importance of sodium reduction for cardiac health, goals of sodium reduction, and 20 sodium reduction strategies; (3) TCM food therapy, including the contribution of TCM to hypertension control, four principles of TCM food therapy, and 34 foods and five herbal teas with antihypertensive functions recommended by TCM food therapy. The intervention delivery consisted of: (1) the DASHNa-CC Intervention Manual; (2) two classroom sessions (2 hours per session); and (3) one 20-minute booster telephone call.

From August to December 2014, 60 participants were recruited in a pilot randomized controlled trial to examine the feasibility and potential effects of the DASHNa-CC intervention. The research findings suggested that participants adhered well to the DASH diet pattern, sodium reduction, and TCM food therapy strategies. The loss to follow-up rate was 5%. Participants were highly satisfied with the intervention and perceived that the intervention contents were helpful, the delivery approaches were suitable, and their participation in the pilot trial was beneficial rather than a burden on their lives. Compared to the control group, the intervention group lowered their systolic blood pressure by 3.8 mmHg (t_55_=–1.58; *P*=.12) more than the control and lowered their diastolic blood pressure by 2.4 mmHg (t_55_=–1.22; *P*=.23). These blood pressure reductions were clinically important to reduce hypertension-related mortality and morbidity [47,48]. In addition, the intervention group had a significant improvement from baseline to eight weeks post-randomization in the physical component score (t_55_=2.13; *P*=.04) of the Medical Outcomes Study 36-Item Short-Form version two (SF-36v2). It is concluded that the DASHNa-CC intervention has the potential to decrease systolic and diastolic blood pressure and improve the health-related quality of life for Chinese Canadians. Three papers, which discuss the main outcome of the DASHNa-CC study [49], the recruitment process [50], and participant satisfaction [51] to the DASHNa-CC intervention, respectively, were published.

### Why is the mDASHNa-CC Smartphone App Needed?

The smartphone app version of the DASHNa-CC intervention (mDASHNa-CC) is proposed because many Chinese Canadian seniors were unable to participate in the pilot study due to busy life schedules (eg, taking care of grandchildren) and were unable to travel to the community center where the DASHNa-CC intervention was delivered. Also, in this pilot study, it was found that most Chinese Canadian seniors were well-educated, technologically savvy, able to access the internet, owned a smartphone, and were willing to learn new skills and knowledge to improve their hypertension control. The academic committee of the DASHNa-CC pilot study suggested transferring the DASHNa-CC to a home-based intervention using a website or smartphone technology to better meet the health care needs of Chinese Canadian seniors. We conducted two focus group discussions with 20 pilot study participants in the community in 2016. All participants had a smartphone and expressed an eagerness to use our app when it becomes available. They stated that they “review the manual frequently, and a smartphone app will make the manual easier to use,” and “this is something new and I want to try.”

### Specific Aims

The overall aim of this two-phase study is to translate the Dietary Approach to Stop Hypertension with Sodium Reduction for Chinese Canadians, a classroom-based antihypertensive dietary educational intervention, to an innovative smartphone app (mDASHNa-CC). This smartphone app would enable and empower Chinese Canadian seniors’ to access this antihypertensive intervention anytime, regardless of where they are.

### Research Objectives and Research Questions

The research objectives are to design and test the usability and feasibility of a smartphone-based dietary educational app to support a healthy diet and hypertension control for Chinese Canadian seniors. In the Phase I usability testing study, the research questions are: (1) How can the mDASHNa-CC app be designed according to the DASHNa-CC intervention and the current literature on hypertension webpages and apps; and (2) How can the mDASHNa-CC app be refined using a user-centered design approach to ensure it is easy to use, efficient, and satisfying for participants? The Phase II pilot feasibility testing study will examine the implementation and preliminary effectiveness of using the mDASHNa-CC app with Chinese Canadians seniors who have hypertension in the community. The research questions of implementation are: (1) What are the rates of participant accrual and attrition; (2) What technical issues arise over the study; (3) What is the acceptability of the study protocol; (4) To what extent do participants adhere to the requirements of learning dietary educational material, conducting dietary self-assessments, and monitoring blood pressure with the mDASHNa-CC app; and (5) To what extent do participants adhere to the dietary recommendations in the mDASHNa-CC app? The research questions of preliminary effectiveness are: Compared to usual care, what is the effect of an 8-week mDASHNa-CC app intervention on systolic and diastolic blood pressure, body weight, waist circumference, health-related quality of life, and health service utilization?

### Theoretical Framework

Social cognitive theory, a behavioral model commonly applied to the design of interventions intended for the self-directed management of chronic disease, will be used as the governing behavior change theory for the mDASHNa-CC intervention. The key constructs of this theory include psychological determinants of behavior, observational learning, environmental determinants of behavior, self-regulation, and moral disengagement [52]. These constructs emphasize the dynamic interaction of personal, behavioral, and environmental factors that could alter human behavior [52]. Social cognitive theory has been successfully used to guide the app design for diabetes control in Canada [53]. Learning from this Canadian evidence, we applied social cognitive theory to our app design for antihypertensive dietary behavior self-management. Evidence suggests that psychological and environmental determinants impact Chinese adults’ dietary behavior and hypertension control. We hypothesized that promoting positive psychological and environmental determinants can enhance dietary behavior and blood pressure control. Learning is considered as the foundational function for the mDASHNa-CC app; therefore, antihypertensive dietary education acts as an essential component of the app. The core function of the app is self-regulation; thus, dietary self-assessments and blood pressure self-monitoring are embedded in this app as the main functions. In addition, automatic feedback according to dietary self-assessments and blood pressure data is incorporated in the app to encourage health behavior changes. This app design will drive self-efficacy through observational learning, self-regulation, and incentive motivation. The behavior changes will be achieved through self-monitoring, tailored feedback, structured education, and incentivizing positive behavior.

## Methods

### Phase I: Design and Usability Testing of the mDASHNa-CC App

Adhering to a phased sequential approach to the development of complex technology-based interventions [54], the development of the mobile Dietary Approach to Stop Hypertension with Sodium (Na) Reduction for Chinese Canadians (mDASHNa-CC) app will include app design, usability testing, and feasibility testing. A user-centered design approach will be applied, where seniors will be actively engaged in all aspects of the research process, including the app’s design, the usability and feasibility testing, and the refinement of the prototype.

Convenience sampling will be used to recruit eligible individuals. To access the most representative sample of Chinese Canadian seniors, a community center in Toronto where Chinese Canadians occupy a high percentage of the total population will be used for blood pressure screening, participant recruitment, app testing, and participant follow-up. In partnership with the Ontario Chinese Senior Association, we will host blood pressure screening events in the Chinese community to facilitate the recruitment process. The app testing procedures will commence after participation eligibility is assessed, informed consent is obtained, and a trained research assistant collects demographic and other outcome data.

This study will include all Chinese Canadians who: (1) are at least 65 years old; (2) have a systolic blood pressure higher than 140 mmHg, or a diastolic blood pressure higher than 90 mmHg, or are on antihypertensive medications, based on preintervention baseline assessment; (3) can understand (listen) and speak in Mandarin or Cantonese, and can read and write in Chinese; and (4) have access to a smartphone. Since self-reporting has been recommended as the preferred approach to measure ethnicity and has been widely applied in public health studies [55,56], the identification of Chinese Canadians in this study will be based on self-reporting of ethnicity. The study will exclude individuals who: (1) have special dietary requirements; (2) are a household member of another mDASHNa-CC participant; or (3) plan to leave the area before the anticipated end of the study.

#### App Design

Based on the findings of the DASHNa-CC pilot trial, the major functions of the app will include: (1) antihypertensive dietary education; (2) dietary self-assessments; (3) automatic feedback according to dietary self-assessments; (4) blood pressure monitoring; and (5) automatic feedback according to blood pressure data. To enhance individuals’ interest in using the app, a new function of the app that will be utilized is built-in age and culture-specific entertainment content as a reward for learning dietary education material, conducting dietary self-assessment, and monitoring blood pressure [57]. The entertainment content, including Chinese songs, videos, or Beijing opera, will be suggested by senior Chinese Canadians in the community.

The antihypertensive dietary education is based on the DASHNa-CC intervention and includes the content of the DASH diet pattern, sodium reduction, and TCM food therapy. It is recommended that participants review the educational content and answer the related questions, and they will also be asked to conduct a dietary self-assessment every day on the app. Based on this, the app will automatically provide feedback and suggestions. Participants are also asked to measure their blood pressure twice a day using a home blood pressure monitor. They will then record the data in the app, which will automatically provide feedback regarding their blood pressure status. Frequent use of the app will be rewarded with built-in entertainment content. The data entered by seniors will be stored locally on the smartphone and then communicated to the server when the phone is online using an encrypted protocol. The server will be hosted at Nipissing University behind a firewall in a secure network environment. A username and password will be required to access the data. The app development will be completed by a software programmer who has extensive experience in educational smartphone app development in the Chinese Canadian community.

#### Usability Testing

Usability testing is a widely used methodology that incorporates an iterative process of testing an intervention’s user-interface and then applying the results to redesign the prototype to meet users’ needs. The current literature supports the importance of usability testing to increase the likelihood of a technology-based intervention’s effectiveness [58]. It is recommended that usability testing takes two to three cycles and involves five to seven participants in every cycle [57,59].

### Low Fidelity User-Centered Design

A qualitative usability testing approach will be used with multiple iterative cycles of semistructured audiotaped interviews. The app interface designs will be trialed with participants. A total of seven seniors in each cycle will be shown paper screenshots of the application and asked what they like and dislike about the interface design, contents, major functions, and built-in entertainment. The list of interview questions will be modified during the interview process considering emerging themes and field notes related to perceived ease of use. The research assistant will record problems with the app. Seniors will also be asked to provide further suggestions for improvement. Design elements will be modified, and new paper screenshots will be generated and tested with iterative cycles until no further changes are suggested.

### High Fidelity User-Centered Design

Following the development of a fully functional smartphone-based prototype, usability testing (multiple iterative cycles) with semistructured audiotaped interviews will be conducted again with seven seniors in each cycle. In this phase, a trained research assistant will first provide seniors with a brief (approximately 5 minutes) demonstration of the app on a smartphone using a standardized dietary assessment vignette. Seniors will then be asked to complete a dietary self-assessment in the app and record their food intake while thinking aloud about their likes, dislikes, and difficulties with the app. At the end of each session, a research assistant will ask a series of open-ended questions related to ease of use, what seniors liked or disliked about the app, and any technical issues. The research assistant will record the answers to questions, write field notes on ease of app use, and explore emerging themes. After the first iterative cycle, changes will be made based on the themes identified from seniors’ opinions. Conflicting suggestions will be handled based on the majority. Another iterative cycle will be conducted with another seven seniors until there are no further recommendations for change to the app.

#### Measurement Tools

The baseline participant demographic characteristics will be collected via the Participant Information Questionnaire. This questionnaire includes 23 questions about socioeconomic status, risk factors for hypertension, and migration history. This questionnaire was used in the DASHNa-CC pilot study. In addition, overall comfort level with smartphones will be ascertained using a questionnaire about smartphone ownership, level of use, and likeability. This questionnaire has been used successfully in previous app studies [57].

#### Data Analysis

Demographic data will be analyzed using the software SPSS version 20.0 (IBM Corporation, New York, United States). To describe the sample, various descriptive statistics (eg, means, standard deviations, proportions), dependent on the level of measurement of the variables, will be calculated for sample demographics and other baseline information.

In both low and high-fidelity usability testing, audiotaped usability interviews will be transcribed verbatim. All transcripts from the usability testing phases will be verified against the tapes and imported into the software NVivo 10.0 (QRS International, Chatstone, Australia) for coding. The field notes taken during the interviews will also be transcribed and included in the analytic process. By using thematic coding, data will be coded according to the study objectives and categorized to reflect the emerging themes [60]. Any changes to the prototype will be made based on feedback from each iterative cycle of testing.

### Phase II: Feasibility Testing of mDASHNa-CC App

Following usability testing, a pilot randomized controlled trial [61] feasibility study will be conducted with Chinese Canadian seniors to determine implementation (primary outcomes, including accrual and attrition rates, technical issues, acceptability of the app, and adherence to the intervention,) and preliminary effectiveness (secondary outcomes, including systolic and diastolic blood pressure, weight, waist circumference, health-related quality of life, and health service utilization). This study is designed as a pilot two-group (1:1) randomized controlled trial with a sample size of 60 participants (block of 20) in a Chinese Canadian community in the Greater Toronto Area.

The sampling procedures and setting will be the same as the Phase I study. A convenience sample of 60 Chinese Canadian seniors will participate in this study. Self-identified Chinese Canadians were recruited if they met the following inclusion criteria: (1) at least 65 years of age; (2) had a systolic blood pressure between 140 to 159 mmHg or a diastolic blood pressure between 90 to 99 mmHg; (3) were able to understand and speak Mandarin and read and write Chinese; and (4) had access to a smartphone. Individuals were excluded if they: (1) used antihypertensive medications, insulin, or oral hypoglycemic agents; (2) had a cardiovascular event during the previous three months; (3) had a history of congestive heart failure; (4) had a cancer diagnosis or had undergone cancer treatment during the past two years; or (5) had special dietary requirements.

As a pilot study is not powered to be a hypothesis-testing trial, formal sample size calculations are not recommended [62]. Instead, the sample size suggested was based on recommendations for feasibility trials [63]. In this pilot study, 60 eligible participants will be recruited. Following university ethics approval, participants will be recruited by blood pressure screening events in diverse community settings. The recruitment process of Phase II is the same as that of Phase I. Study procedures will commence after eligibility is assessed, informed consent is obtained, and demographic and baseline outcome data are collected by a trained research assistant.

Using an online randomization tool provided by Interrand Company, Ottawa, Canada, participants will be randomized into either an intervention group or a control group. Participants randomized to the intervention group will receive the mDASHNa-CC app intervention for eight weeks plus usual care; participants randomized to the control group will receive usual care. Coinvestigators, collaborators, and outcome assessors will be blinded to the group assignment. Because this study is an app educational intervention, it will be impossible to blind participants to the group assignment. The group assignment will be concealed until all outcome data are collected [64]. After all outcome data are collected eight weeks post-randomization, participants in the control group will also be offered use of the app.

The control group will be usual care. Usual care consists of three parts: (1) receiving a general hypertension health education booklet from the Heart and Stroke Foundation of Ontario; (2) being encouraged to see their family physicians or primary health care providers regarding their blood pressure status (those who do not have a primary health care provider will be referred to a walk-in clinic or a community health center); and (3) having access to family physicians, telehealth, emergency care, hospitals, and other health care facilities in the Greater Toronto Area as required.

In addition to usual care, those participants randomized to the intervention group will be offered use of the app. A trained research assistant will teach them how to use it, they will load the app on their smartphones, and then they will be requested to review educational material, conduct dietary self-assessments, and monitor blood pressure for eight weeks. Telephone assistance from a trained research assistant will be available to seniors in case of technical problems or if any questions arise about the app. The research team will conduct a daily review of a summary of each senior’s report so that participant safety issues can be identified and resolved. By the end of the eight weeks postrandomization, seniors will be prompted by phone using an audible alert to complete the app evaluation questionnaire on their smartphone, which will ascertain likes and dislikes with the app.

#### Measurement Tools

Baseline demographic characteristics will be collected via the participant information questionnaire, which is described in the measurement tools section in the Phase I study. Implementation outcomes will describe the feasibility of using the app with Chinese Canadian seniors in the community. Implementation will be measured as:

- Accrual and attrition rates: The mDASHNa-CC recruitment log has been designed to record data related to the number of eligible seniors per recruitment day, reasons for ineligibility, and reasons for nonparticipation. The mDASHNa-CC Activity Log has been designed to record data on attrition, including occurrence/reasons for attrition, technical difficulties, adherence, and outcome measure completion. A trained research assistant will complete the logs daily during the research process.
- Technical issues: The occurrence and description of technical problems will be recorded on the mDASHNa-CC activity log by a trained research assistant.
- Acceptability: The acceptability e-scale ascertains perceptions related to how helpful, difficult, and enjoyable electronic-based programs are to use, how understandable questions are, and how acceptable the time invested in reporting was [65]. This scale demonstrated validity and reliability in various prior studies [65]. For the present study, the wording of the scale will be slightly modified, and a free-text question, where seniors are encouraged to enter any other information that they feel would be important to discuss, will be added. Seniors in the intervention group will fill out this scale by email four weeks and eight weeks postrandomization.
- Adherence: A built-in number counter will measure participants’ adherence to the requirements of learning dietary educational material, conducting dietary self-assessments, and monitoring blood pressure in the mDASHNa-CC app. The number counter will record the time and frequency of app use. The dietary intake will be measured at baseline and eight weeks postrandomization by a one-to-one dietary interview using the validated Automated Multiple Pass Method [66] by a trained research assistant in the community center. This approach was successfully tested in the DASHNa-CC pilot trial. Adherence to the DASH will be measured by the validated DASH component score of each food group, and the total DASH score [67]. Adherence to the sodium reduction will be measured by a 24-hour urine test [68]. Adherence to TCM food therapy will be measured using a 24-statement questionnaire on a 5-point Likert scale, which was validated in the DASHNa-CC pilot study [69].

#### Preliminary Effectiveness and Outcomes

Except for health service utilization, which will be measured only at eight weeks postrandomization, the other following outcomes will be measured at baseline and eight weeks postrandomization at the community center by a trained research assistant during a one-to-one appointment. If a participant cannot visit the center, a home visit by a trained research assistant will be arranged.

- Systolic and diastolic blood pressures: Systolic and diastolic blood pressures will be measured with the home blood pressure monitor, Omron BP785, whose validity and reliability have been tested [70]. All blood pressure measurements will be performed following the recommended techniques by the Canadian Hypertension Education Program guidelines [5]. Each participant will be offered an Omron BP785 and training on how to measure blood pressure at home and how to record the results; however, blood pressure at baseline and eight weeks postrandomization will be measured by a trained research assistant.
- Bodyweight: An electronic body weight scale will measure weight.
- Waist circumference: Waist circumference will be measured by measurement tape following proper techniques [71-74].
- Health-related quality of life: Health-related quality of life will be measured by the SF-36v2 [75,76].
- Health service utilization: Health service utilization data will be collected via the Health Service Utilization Questionnaire, which was modified from the Health Service Utilization Questionnaire previously used in Ontario [77].

### Data Analysis and Statistical Methods

Demographic data will be analyzed using the software SPSS version 20.0 by a biostatistician. To describe the sample, various descriptive statistics (eg, means, standard deviations, proportions), dependent on the level of measurement of the variables, will be calculated for sample demographics and other baseline information. Descriptive statistics will be calculated to demonstrate how participants adhered to and are satisfied with the app. Open-ended questions will be reviewed by two researchers independently. Qualitative data will be organized into meaningful groups, combining similar patterns into themes. In feasibility testing, adherence is defined as 100% when 28/28 blood pressure entries and 14/14 dietary self-assessments entries are completed within the two weeks. An independent, two-sample, two-tailed *t* test will be used to examine the differences in mean change scores between the control and intervention groups regarding blood pressure, weight, waist circumference, and health-related quality of life.

### Quality Control

Quality control strategies will be implemented. Onsite training will be provided to the research staff. The training will include research ethics, privacy and confidentiality, orientation to research protocol and procedure, literature search and review, data collection tools, data analysis methods, and community networking. The training will provide staff with adequate time for interactive learning, on-site practice, and skill preparation for comprehensive teamwork in the project [78]. To make sure there is consistency in data collection, a uniform data collection tool will be used. The principal investigator will work closely with research staff in the data collection and analysis process. Team meetings will be conducted every month for progress updates and problem-solving. Research staff will be requested to make research notes daily. Discussion and debriefing will be provided promptly if needed. To sustain participant motivation to the project, patient contact will be organized in patient-preferred time to encourage participation. A research assistant will remind participants in advance of each research event [79]. In addition, city public transport tickets will be provided to assist with commuting costs. Participation certificates will be provided to honor participants’ contributions to the study.

## Results

The study is supported by the Startup Research Grant from Nipissing University, Canada. The research ethics application is under review by a university research ethics review board.

## Discussion

### Knowledge Translation

Our knowledge translation plan incorporates strategies to ensure that our app will stand apart from existing apps in the eyes of Chinese Canadian seniors and other Chinese populations. Firstly, our networks with Chinese Canadian communities and our engagement of key consumer groups (Ontario Chinese Senior Association and other Chinese senior groups and community centers) will help to spread awareness of our app to people in the community using a grassroots approach. Collaboration with the community to provide workshops, information sessions, support groups, and social media interviews will promote the use of our app in the community. Secondly, our research team includes a nurse, a dietitian, a TCM practitioner, and a medical doctor who are in various organizations in Canada. Upon completion of the project, these leaders will be able to endorse the uptake of our app at their clinics and the practices of their colleagues. Thirdly, we will enter our app into competitions for technology design awards nationally and internationally to further solidify its credibility and earn patient buy-in. This strategy can also help disseminate our app outside Canada, including the United States, where 2.4 million Chinese individuals live, and East Asian countries, such as China, where hypertension is emerging as a critical public health issue [80,81]. Fourthly, we will partner with Hypertension Canada, Toronto Public Health, Wenzhou Medical School (P. R. China), and other organizations to promote the use of our app. Endorsement of an app by health promotion organizations can lend legitimacy to a new tool. Fifthly, national, and international academic audiences will be reached through academic publications and presentations at conferences by researchers. Finally, fact sheets, research summaries, presentations in leadership forums, and policy recommendations will be used to communicate research findings with government and policymakers to promote related policy changes.

### Human Subjects

Chinese Canadian seniors will be the research subjects in this study. As a technology-based dietary educational intervention, this study poses no known risks to participants. All participants have access to telehealth, emergency care, hospitals, and other health care facilities in the Greater Toronto Area. All participants are free to use all these health care services anytime, as needed.

There are no known benefits to participation in this pilot trial. However, participants in the DASH trial reported reduced blood pressure and enhanced quality of life [15,82]. Participants in a trial of TCM food therapy in China also reported improved health-related quality of life and reduced the use of their antihypertensive medications [45]. By participating in this study, participants will gain knowledge about healthy eating and the importance of blood pressure control. Participants will be instructed to monitor their blood pressure. In addition, a CAD$20 ($15) dollar gift card and a certificate of participation will be offered to all participants as a token of appreciation for their participation. Every participant will be offered two city public transportation tickets every time they attend the research activities to compensate for travel expenses. Participants will receive the study results by email.

### Implications

The study results will make contributions in six areas: (1) produce a smartphone app, which allows a large number of seniors, their families, and other community members to access the dietary intervention for hypertension control, and could potentially be used across Canada and internationally in large Chinese ethnic populations; (2) illustrate the rigorous design and testing of smartphone app technology for hypertension self-management in the community; (3) explore the approach of incorporating traditional medicine in chronic illness management in minority communities; (4) contribute to culturally sensitive care, which is an urgent need due to global migration and has implications for immigrant-recipient countries and multiethnic societies; (5) promote equal access to current technology among minority immigrant senior groups; and (6) facilitate the full randomized trial in the future to examine the effects of the app on blood pressure and health-related quality of life.

## Abbreviations

DASH: Dietary Approach to Stop Hypertension
DASHNa-CC: Dietary Approach to Stop Hypertension with Sodium (Na) Reduction for Chinese Canadians
mDASHNa-CC: Mobile Dietary Approach to Stop Hypertension with Sodium (Na) Reduction for Chinese Canadians
SF-36v2: Medical Outcomes Study 36-Item Short-Form version two
TCM: Traditional Chinese Medicine
